# Oral health needs of U.S. children with developmental disorders: a population-based study

**DOI:** 10.1186/s12889-022-13237-2

**Published:** 2022-04-29

**Authors:** Raghad Obeidat, Amal Noureldin, Anneta Bitouni, Hoda Abdellatif, Shirley Lewis-Miranda, Shuling Liu, Victor Badner, Peggy Timothé

**Affiliations:** 1 Public Health Sciences Texas A&M University College of Dentistry, 3302 Gaston Avenue, Dallas, TX 75246 USA; 2 Department of Comprehensive Dentistry, Texas A&M University College of Dentistry, 3302 Gaston Avenue, Dallas, TX 75246 USA; 3 Princess Nourah Bint, Abdulrahman University, Riyadh, Saudi Arabia; 4 Statistical Collaboration Center, Texas A&M University, 155 Ireland Street, College Station, TX 77843 USA; 5 Depts. of Dentistry and Epidemiology and Population Health, Albert Einstein College of Medicine, Jacobi Medical Center, 1400 Pelham Parkway South, Bronx, New York City, NY 10461 USA

**Keywords:** Children with developmental disorders, Developmental disabilities, Oral health, Access to health care, Barriers to dental care

## Abstract

**Background:**

Children with Special Health Care Needs (CSHCN) have higher rates of oral diseases and tooth decay compared with the general population. Children with developmental disorders/ disabilities (DD) are a subset of CSHCN whose oral health has not been specifically addressed**.** Therefore**,** this study had two objectives: to describe the oral health needs (OHN) of children with DD compared with children without DD; and to assess barriers to access to care, utilization of dental services, and their association with oral health needs for children with DD.

**Methods:**

This cross-sectional study utilized a sample of 30,530 noninstitutionalized children from the 2018 National Survey of Children’s Health (NSCH). Analysis was conducted using descriptive and inferential statistics.

**Results:**

The analysis identified 6501 children with DD and 24,029 children without DD. Children with DD had significantly higher prevalence of OHN (20.3% vs. 12.2%, respectively), unmet dental needs (3.5% vs 1.2%), and utilization of any dental visits (86.1% vs 76.1%), (*P*-value < . 001). The adjusted logistic model identified four factors that contributed to the higher odds of OHN among children with DD: poverty (< 100% of the Federal Poverty Level (AOR = 2.27, CI: 1.46–3.51), being uninsured (AOR = 2.12, 95% CI: 1.14–3.95), a high level of disability (AOR = 1.89, CI: 1.23–2.78), and living in the western United States (AOR = 1.61, CI: 1.09–2.37.

**Conclusion:**

Despite higher utilization of dental services, children with DD had poorer oral health and more unmet dental needs than children without DD. Advocacy efforts and policy changes are needed to develop affordable access that assesses, as early as possible, children with DD whose conditions impact their ability a great deal so that their potential OHN may be alleviated more effectively.

## Background

The Maternal and Child Health Bureau (MCHB) defines children with special health care needs (CSHCN) as “those who have or are at increased risk for a chronic physical, developmental, behavioral, or emotional condition and who also require health and related services of a type or amount beyond that required by children generally” [1]. Studies in the literature reported a significant burden of oral diseases among CSHCN [2–4]. High caries risk and caries burden were reported in a 2019 study conducted in North Carolina (*n* = 150) measuring caries risk among different groups of CSHCN [2]. In another study that described the oral health status among CSHCN using 1128 completed surveys of families of CSHCN throughout urban and rural Massachusetts, Nelson et al. found that the oral health status of 20% of the study’s sample was reported as fair or poor [3]. Iida et al. using the data of 9,936 children younger than 18 years from the 2005 Medical Expenditure Panel Survey (MEPS) found higher unmet dental care needs for CSHCN compared with children in general, especially teenagers, children in poverty, children who were uninsured or had insurance gaps, and those who were severely affected by their conditions [4].

Access to healthcare and its related factors are also reported to influence the oral health of CSHCN. According to the National Academy of Medicine (NAM), access to healthcare is an umbrella term. It is measured by three indicators: barriers (structural, financial, and personal), utilization (visits and procedures), and outcomes (health outcomes and equity) [5]. Utilization of health services is often reported among healthcare-related factors that influence oral health among CSHCN [6–8]. Craig et al. found that CSHCN enrolled in Medicaid within Washington state’s Access to Baby and Child Dentistry (ABCD) program (*n* = 206,488) were less likely to use preventive dental care than children without SHCN [9].

Sarkar et al. using the data from the 23,000 Ohio residents of the 2012 Ohio Medicaid Assessment Survey (OMAS) found that CSHCN enrolled in Medicaid had more unmet dental needs and were less likely to have excellent oral health than CSHCN with private insurance [8]. Using the data from the National Survey of Children with Special Health Care Needs, Sannicandro et al. [10] compared the health care utilization of children with special health care needs in 2005/06 (*n* = 40,723) and 2009/10 (*n* = 40,242) and found that CSHCN encountered barriers to obtain dental care and had unmet dental needs. CSHCN who had moderate (OR = 1.74, *p* < 0.001) and consistent disability (OR = 2.30, *p* < 0.001) were more likely to have unmet dental needs. CSHCN were more likely to have unmet dental needs if they live with one biological and one stepparent (OR = 1.42, *p* < 0.01), live in a single-parent household (OR = 1.29, *p* < 0.01), or live in a household with no health insurance (OR = 3.74, *p* < 0.001). Unmet dental needs were also associated with poverty. CSHCN were less likely to have unmet dental needs if they live in households between 200 and 399% of the federal poverty level (OR = 0.68, *p* < 0.001) or above 400% of the federal poverty level (OR = 0.33, *p* < 0.001).

Research also found that unmet needs were higher in regions with greater health professional shortage areas and in regions with greater poverty [11]. Paschal et al. assessed regional differences for unmet dental needs using 2009–2010 National Survey of CSHCN (*n* = 40,242) found that those who live in the West region were more likely to have more unmet needs for preventive and specialized dental care than in the reference region (Northeast). The South region followed the West region [11].

Individual-level factors for oral diseases have been found to play an important role in poor oral health among CSHCN. These include a diet high in sugar, dependence on caregivers for oral hygiene, and sugary medications or medications that impair saliva’s excretion [3, 7, 12].

Children with developmental disorders/disabilities (DD) are a subgroup of CSHCN who have various physical, behavioral, and cognitive limitations that affect their abilities to perform activities of daily living, including maintaining their oral health [13]. Dental treatments for these children are challenging due to multiple factors including communication, behavior and cooperation with the provider. Improving the oral health and meeting the needs of children with DD is important to improving the quality of life of these vulnerable populations and reducing the burden on their families and the society [6, 12, 14].

There have been studies on the oral health of CSHCN populations, but literature on the subset of children with DD is sparse. Most of the literature addressed only the oral health of children as a broad group of CSHCN with its heterogeneity of health conditions or with individual disorders such as Autism Spectrum Disorders (ASD) and Down Syndrome (DS). Our study adds to the literature by reporting on this important subset of CSHCN, with a focus on the oral health challenges faced by children with DD and their respective caregivers.

Children with DD often face more challenges in obtaining health care, especially dental care [6, 15, 16]. Several studies identified barriers of access to dental care among children with DD [17–19]. However, the extent to which these barriers impact oral health of children with DD remains unknown. Mindful of the increasing prevalence of DD in children [20], we undertook this study to investigate the impact of access to healthcare-related factors on the OHN of children with DD at the national level. This study had the following objectives: 1) to describe the oral health needs (OHN) of children with DD compared with children without DD; and 2) to assess barriers to access to care, utilization of dental services, and their association with oral health needs for children with DD.

## Methods

This study was conducted from December 2019 through June 2020. The National Survey of Children’s Health (NSCH) data for the year 2018 were employed. NSCH is a screening for various developmental disorders that provides data on different, intersecting aspects of children’s lives including physical and mental health, parental health, access to health care, family, and social environment [21]. Of the 176,052 sampled addresses in the 50 states and the District of Columbia, NSCH included completed interviews of a parent or other caregiver of a representative national sample of 30,530 of non-institutionalized children aged 0–17 years and 520–796 participants per individual state [22]. The survey was conducted as a mail and web-based survey administered by the Data Research Center for Child and Adolescent Health (DRC) in partnership with the MCHB and the U.S. Census Bureau. A weighted overall response rate of 43.1% was achieved. NSCH data are publicly available on the Census Bureau’s NSCH page. Further information on sample methodology and selection may be found on the DRC website (childhealthdata.org).

We identified children with DD based on the definition established by the American Academy of Pediatrics (AAP) [22, 23]. The child was included in the DD group if he/she had any or a combination of the following: Autism Spectrum Disorders (ASD), Down Syndrome (DS), Attention Deficit Disorders (ADD/ADHD), Cerebral Palsy (CP), Intellectual Disability (ID), epilepsy, Tourette syndrome, developmental delay, learning disability, behavioral and conduct disorders, and speech disorder. We determined that 6,501 children met this definition.

### Study variables

We utilized the model of access to healthcare by the NAM [5]. Thus, we included the following indicators in our theoretical framework (see Table 1): barriers of access to healthcare (personal, financial, and structural); utilization of dental services; and outcomes variables (OHN and unmet dental needs).

**Table 1:** Study variables

Variable Type	Variable Name	Type	Subtype
**Independent variables**	**Barriers to healthcare access**		
		Structural barriers	residence (metro/non-metro), census bureau regions
		Financial barriers	health insurance coverage, health insurance type, Federal Poverty Level (FPL)
		Personal barriers	extent of disability
	**Utilization of dental services**		
		Annual dental provider visit	
		Annual preventive visit	
**Dependent variable**	**Outcomes**		
		Oral health needs (OHN)	
		Unmet dental needs	
**Covariates**	age, race/ethnicity, family structure, guardian education, household language		

### Ethics review

The Institutional Review Board of Texas A&M University determined that this project “is not research involving human subjects as defined by DHHS and FDA regulations.” The IRB added: “Further IRB review and approval by this organization is not required because this is not human research.” (Correspondence: IRB2020-1004; 9/14/2020).

### Utilization of dental services

Specifically, the utilization of dental services was analyzed using questions regarding annual dental provider visits and annual preventive visits in the NSCH. Any annual dental provider visit was further collapsed into two groups: “Yes, saw a dental provider” and “No, did not see a dental provider during the past 12 months.” For annual preventive visit, we used the survey’s question: “During the past 12 months, if a child saw a dental provider for preventive dental services such as check-ups, cleaning, sealants, and fluoride treatment?” We classified the children into two groups: “No, did not see a dental provider for a preventive visit” and “Yes, saw a dental provider once or twice within the past 12 months.”

### Barriers to access to oral healthcare

In terms of structural barriers, two variables were used for geographic location: residence (metropolitan and non-metropolitan) and Census Bureau regions. A Metropolitan Statistical Area is defined by the U.S. Office of Management and Budget as containing an urbanized area with a population of at least 50,000 [24]. In the NSCH, since child’s state of residence was collected as Federal Information Processing Standard (FIPS) State Code, we created four categories for the Census Bureau regions: Northeast (Connecticut, Maine, Massachusetts, New Hampshire, New Jersey, New York, Pennsylvania, Rhode Island, and Vermont); Midwest (Illinois, Indiana, Iowa, Kansas, Michigan, Minnesota, Missouri, Nebraska, North Dakota, Ohio, South Dakota, and Wisconsin); South (Alabama, Arkansas, Delaware, District of Columbia, Florida, Georgia, Kentucky, Louisiana, Maryland, Mississippi, North Carolina, Oklahoma, South Carolina, Tennessee, Texas, Virginia, and West Virginia); and West (Alaska, Arizona, California, Colorado, Hawaii, Idaho, Montana, Nevada, New Mexico, Oregon, Utah, Washington, and Wyoming) [25].

For the financial barriers, since no question was asked about dental insurance, “health insurance coverage within the past 12 months” was used as a proxy and includes two categories: insured all 12 months and uninsured all 12 months. The health insurance types were further divided into four categories: public, private, public and private, and uninsured. Four categories for the Federal Poverty Level (FPL) were used to indicate income/poverty level: 0–99%, 100–299%, 300–399%, and 400% and above.

For the personal barriers, we measured the extent of disability which was developed from parents’ responses to two questions in the NSCH: “Health condition affected ability- How often” and “Health condition affected ability -Extent”. Ability was defined as the child’s ability to do things other children his or her age do. If parents responded that their child’s health condition had no impact on his/her ability, the child was categorized as “never” for the extent of the disability. If they responded as “yes” the health condition affected their child's ability somehow, they were asked to describe the extent into three categories: very little, somewhat and a great deal. Accordingly, the extent of the disability variables included four groups: never, very little, somewhat, and a great deal.

### Dependent variable

Our dependent variable is the perceived OHN, which is a dichotomous variable that we developed from parents’ responses when asked if their child had any of the following oral conditions during the past 12 months: cavities, bleeding gum, and/or toothache. If the parents’ response was “yes” to any of these conditions, the child was classified as having OHN. The other outcome variable, unmet dental needs, was developed from parents' responses to the question: “During the past 12 months, was there any time when this child needed healthcare, but it was not received?”. If parents’ response was “yes”, parents asked to choose from a list of health care services (medical, dental, mental, hearing, and vision) that a child needed but had not received. However, we did not use unmet dental needs as a dependent variable for bivariate and logistic regression as conducted for OHN because in our prospective, the literature is definitive on the unmet dental needs for CSHCN. However, oral health status measured by OHN rarely were addressed in the literature especially for children with DD as a subpopulation.

### Covariates

Additionally, covariates such as age, race/ethnicity, family structure, guardian education, and household language were developed from items present in the NSCH. Age was developed from a continuous variable (0–17) into three categories based on a phase of dentition: < 6 years old (primary), 6–12 years old (transitional), and 13–17 years old (permanent). Race/Ethnicity was developed from two variables, race and ethnicity, to provide five racial/ethnic categories: Whites, African Americans or Blacks, Hispanics, Asians, and Others. Family structure was collapsed into three categories: two parents, single mother, and others. Guardian education included two categories: less than high school or high school and some college or higher. Household language was classified into 2 groups: English and non-English.

### Statistical analysis

Data were analyzed with IBM SPSS software, version 26. Descriptive statistics and bivariate analysis (Chi-square test) were used to compare oral health status, unmet dental needs, and utilization of dental services between children with and without DD. Additionally, frequency tables were used to summarize sociodemographic factors and factors related to access to health care for our sample of children with DD stratified by OHN status. Multi-variable logistic regression analysis was conducted to examine the association between OHN and each variable related to access to healthcare. We checked for collinearity between variables using the Variance Inflation Factor (VIF) and we conducted variables’ selection model.

To ensure proper variance estimation, statistical estimates were calculated for the complex sample design (to adjust clustering, stratification, and non-response). For the analysis, all variables were weighted to represent the population of non-institutionalized children 0–17 nationally. The child’s weight was composed of a base sampling weight, adjustments for both screener and topical nonresponse, an adjustment for the selection of a single child within the sample household, and adjustments used to control to population counts for various demographics obtained from the 2017 American Community Survey (ACS) one-year data. All percentages, confidence intervals (CI), and p values reflect the sampling weights and are thus generalizable to nationally representative estimates. Adjusted Odds Ratio (OR) and 95% CI were reported.

## Results

We found that children with DD were more likely to be males (64.1%); school-age children (66.3%); Whites (53.1%); living with guardians who had some college or more education (69.5%); English speaking (91.8%); living in a two-parent family (74.0%); in a household with income above 200% FPL (55.3%); living in metropolitan areas (73.7%); and residing in the South region (40.4%). More than half of them (58.5%) had not been affected by their condition.

### Oral health status, oral health needs, and unmet needs

**Fig. 1:**
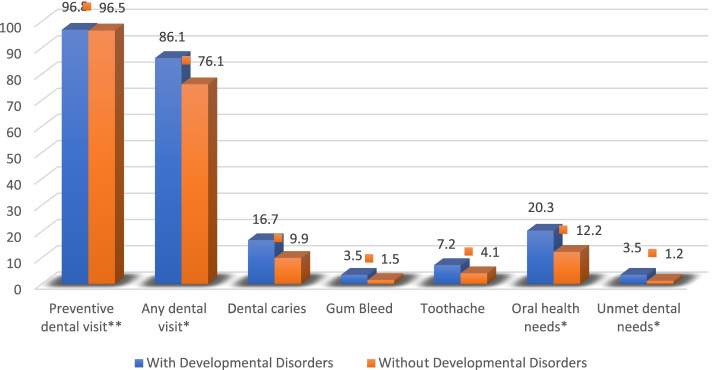
Children’s utilization of dental services, oral diseases, OHN, and unmet dental needs, stratified by developmental disorders status, *n* = 30,530 *P < .000 each comparison between children with and without DD: any dental visit, oral health needs, and unmet dental needs. **P = .639 for preventive dental visit

In terms of oral health status as reported by parents, dental caries are the most prevalent oral diseases among our sample. The prevalence of caries is 16.7% among children with DD compared with 9.9% for children without DD. The prevalence of bleeding gums is 3.5% among children with DD and 1.5% among children without DD. Moreover, the prevalence of toothache is 7.2% among children with DD and 4.1% among those without DD. A significantly higher proportion of children with DD relative to children without DD were found to have OHN (20.3% vs. 12.2%, respectively, *P* < 0.000; Fig. 1). Furthermore, 3.5% of children with DD compared to 1.2% of children without DD reported having needed health care that was not received (unmet dental need) (Fig. 1). Although the rate of unmet dental needs is relatively low, it is more than twice that for children with DD compared with children without DD.

A higher proportion of children with DD relative to children without DD was found to utilize any dental services in the past 12 months (86.1% vs 76.1% *P* < 0.000) (Fig. 1). However, there was no difference for preventive dental preventive visits between those with and without developmental disorders (96.8% vs. 96.5%, *P* = 0.639).

For our sample of children with DD, our bivariate analysis shows no association between OHN and any dental provider visit (86.9.1% vs 85.9.1%, *P* = 0.643) nor preventive dental visit (96.9% vs 96.7%, *P* = 0.866) (Table 2).

**Table 2:** Characteristics of children with DD stratified by parent’s reported oral health needs status, *n* = 6501

	**All % (weighted)**	**With oral health needs 18.3%** **(weighted)**	**Without oral health needs 81.5% (weighted)**	***P*- value**
**Characteristics of child**				
Sex of child				.229
Male	64.1	67.1	63.4	
Female	35.9	32.9	36.6	
Age of Child				**.000**
< 6 years old (primary dentition)	16.4	11.3	17.7	
6–12 years old (transitional dentition)	49.9	60.6	47.2	
13–17 years old (permanent dentition)	33.7	28.1	35.2	
Race of child				.603
White	53.1	50.9	53.7	
African American or Black	14.7	14.3	14.8	
Hispanics	23.3	26.5	22.5	
Asian	2.3	2.6	2.3	
Other	6.5	5.8	6.7	
Primary language				.122
English	91.8	88.6	92.6	
Non-English	8.2	11.4	7.4	
**Family/ Household Characteristics**				
Guardian education				**.000**
Less than high school or high school	30.5	39.5	28.2	
Some college or more	69.5	60.5	71.8	
Family Structure				.151
Two parents	74.0	70.0	75.0	
Single mother	22.7	26.4	21.7	
Other	3.3	3.6	3.3	
Federal Poverty/income level				.000
0–99% federal poverty level	20.9	31.0	18.4	
100%–199% federal poverty level	23.7	26.8	22.9	
200%–399% federal poverty level	26.5	23.1	27.4	
400% federal poverty level or above	28.8	19.1	31.3	
Residence				**.008**
Metro	73.7	70.2	74.6	
Non-Metro	11.5	15.6	10.5	
Non-disclosed	14.8	14.2	14.9	
Census Bureau Regions				.389
Northeast	16.0	14.4	16.5	
Midwest	20.7	19.0	21.1	
South	40.4	40.5	40.4	
West	22.8	26.1	22.0	
Any Dental Visit	86.1	86.9	85.9	.643
Preventive dental visit	96.8	96.9	96.7	.866
Disability extent				**.001**
Never	58.5	48.9	61.0	
Very little	11.8	13.6	11.3	
Somewhat	21.2	25.0	20.2	
A great deal	8.5	12.5	7.4	
Health Insurance Coverage (Past 12 months)				**.001**
Insured	91.4	86.6	92.7	
Uninsured	8.6	13.4	7.3	
Health insurance Type				**.000**
Private	51.0	38.1	54.3	
Public	36.8	46.3	34.4	
Public and private	6.5	8.0	6.1	
Not insured	5.6	7.6	5.2	

^*^All percentages are weighted

^**^Cross tabulation of OHN with child and family characteristics, utilization of dental services, and access to healthcare barriers

### Barriers to oral health for children with DD

For structural barriers, differences existed in OHN among children with DD by residence location: 70.2% of children with DD with OHN lived in metropolitan areas versus 74.6% without OHN. In non-metro areas, we found 15.6% with OHN versus 10.5% without OHN (*P* < 008). Residence by Census Bureau region was not significantly associated with OHN (*P* = 0.389). Of the four regions, the South accounted for the most children with DD with OHN (40.5%); the Northeast had the fewest (14.4%). However, children with DD who live in the West had a higher proportion of OHN (26.1% with OHN vs. 22.0% without OHN). In contrast, the Midwest had a lower proportion of children with DD with OHN (19.0% with OHN vs 21.1% without OHN).

For financial barriers, we found a statistically significant difference for health insurance coverage between children with DD with and without OHN. For children with DD with OHN, 86.6% were insured the entire past 12 months, compared with 92.7% for children with DD without OHN (*P* < 0.001). Children who were uninsured the entire past 12 months were more likely to have OHN. The type of health insurance was also significantly associated with OHN (*P* < 0.001). Children with DD with private insurance had a lower proportion of OHN compared to children with public insurance (38.1% vs 46.3%). Furthermore, children with DD with both private and public health insurance were more likely to have OHN (8.0% with OHN vs 6.1% without OHN). A significant difference was also found for income levels between children with DD with and without OHN (*p* < . 001). Among children with DD with OHN, 80.9% were below the 400% FPL compared with 68.7% for children with DD without OHN.

For personal barriers, children with DD were classified according to their ability to do things most children of the same age usually do: never affected; affected very little; affected somewhat; and affected a great deal. The results suggested that the more children with DD are affected by their condition, they were more likely to meet the OHN criteria. Specifically, children with DD who are never affected by their condition accounted for 58.5% of children with DD but only 48.9% of those with OHN (*p* < 0.001). Children with DD whose conditions affected their ability a great deal accounted for 8.5% of children with DD but 12.5% of those with OHN (*p* < 0.001).

When we examined the association between OHN among children with DD and various potential predictive variables, including sociodemographic variables, through multivariable regression analysis (Table 3), we found that elementary school children (aged 6–12 years) had higher adjusted odds of OHN (AOR: 1.88, 95% CI: 1.21–2.93). We also found that children living in the West region had a statistically significant higher odds of OHN than those living in the Midwest (AOR: 1.61, 95% CI: 1.09–2.37).

**Table 3:** Adjusted multiple logistic regression for the association between OHN and child/family characteristics and access to healthcare barriers among children with DD

Variable	Point Estimate	95% CI
Disability severity		
Never	Referent	
Very little	1.48	.99 – 2.21
Somewhat	**1.43**	**1.06 – 1.94**
A great deal	**1.89**	**1.23 – 2.78**
Health Insurance Coverage (past 12 months)		
Insured	Referent	
Uninsured	**2.12**	**1.14– 3.95**
Health insurance Type		
Private	Referent	
Public	1.16	.82 – 1.62
Public and private	1.33	.86 – 2.05
Not insured	.66	.23 – 1.51
Poverty income level		
0–99% federal poverty level	**2.27**	**1.46 – 3.51**
100%–199% federal poverty level	**1.58**	**1.07– 2.33**
200%–399% federal poverty level	**1.44**	**1.01 – 2.04**
400% federal poverty level or above	Referent	
Census Bureau Regions		
Northeast	1.18	.77 – 1.81
Midwest	Referent	
South	1.11	.83– 1.50
West	**1.61**	**1.09 -2.37**
Residence		
Metro	Referent	
Non-Metro	**1.42**	**1.02 – 1.99**
Non-Disclosed	.96	.71 – 1.29
Characteristics of the child/Parents		
Age		
< 6 years old	Referent	
6–12 years old	**1.88**	**1.21– 2.93**
13–17 years old	1.22	.78– 1.90
Race/Ethnicity		
White	Referent	
African American or Black	.68	.45 – 1.06
Hispanics	.84	.55 – 1.26
Asian	1.19	.59 – 2.41
Other	.66	.47 – .95
Guardian Education		
Less than high school or high school	1.18	.87– 1.61
Some college or more	Referent	
Household language		
English	Referent	
Non-English	1.21	.61 – 2.42
Family structure		
Two parents	Referent	
Single mother	1.10	.82– 1.48
Other*	1.14	.63 – 2.08

^*^ Other include single father, grandparent household, and other relation , **Bold indicates significance**

Children who lived in households with income less than or equal to 400% FPL had higher adjusted odds of OHN than those who lived in households with income greater than 400% FPL. A statistically significant trend was found for higher adjusted odds of OHN with an increasing level of poverty (*P* < 0.000). Children with DD who were uninsured had higher odds of OHN than children with DD who were insured the entire past 12 months (AOR = 2.12, 95% CI: 1.14–3.95). However, for children with DD who had public health insurance or both public and private health insurance, the results were not statistically significant. Lastly, children with DD who had been affected by their conditions a great deal had higher adjusted odds of OHN than those who had been affected somewhat by their condition (AOR = 1.89, 95% CI: 1.23–2.78 and AOR = 1.43, 95% CI: 1.06–1.94, respectively).

## Discussion

This study is the first to investigate the impact of barriers to access to dental healthcare on the OHN of a representative sample of U.S. children with DD at the national level. Overall, we found that children with DD had higher OHN and unmet dental needs compared with children without DD. However, use of dental services as measured by dental visits was found not associated with OHN among children with DD. Poverty, health insurance coverage, urbanicity, residence by census regions, and the level of disability are barriers found to be associated with OHN. Our adjusted logistic model found that being uninsured, poor (< 100% FPL), and having a great deal of disability severity had the greatest impact on the OHN among children with DD.

Dental caries and periodontal diseases are prevalent among U.S. CSHCN [2, 3, 26–28]. Our findings of a higher prevalence of parent-reported oral diseases among children with DD compared with children without DD are consistent with most of the studies in the literature that investigated oral health status among CSHCN.

### Utilization of dental health services and unmet dental needs

Our findings of the high use of any dental services among children with DD were consistent with the finding of Iida et al., who found that CSHCN used more dental care services and were more likely to receive only non-preventive care than children without special healthcare needs (4). This was also confirmed by our finding of a non-significant difference for the use of preventive dental care between children with DD compared with children without DD. There was no significant association between OHN and either any dental visit use or preventive dental visit use among our sample of children with DD. This finding was consistent with the results of Nelson et al. and Iida et al. regarding utilization of dental services for CSHCN [3, 4].

The rate of unmet dental needs among children with DD was nearly three times that among children without DD. We also found that the rate of unmet needs among children with DD at the national level (2.4%) was lower than the rate of unmet needs (20%) of the study conducted by Nelson et al., which was limited to Massachusetts [3]. Moreover, our unmet needs rate among children with DD (2.4%) was also lower than the rate of unmet dental needs among CSHCN (8.9%) that was reported by Lewis et al. [7] using the 2006 NSCH. The discrepancies could be explained by the improvement made in meeting the needs of CSHCN such as services offered through Title V Maternal and Child Health Services Block Grant Program for CSHCN [29].

We also investigated the association with OHN among children with DD for the following barriers: geographic location (structural), health insurance and poverty (financial), and the extent of disability (personal).

### Geographic location

Although a higher number of children with DD was found in the South, children living in the West had the highest proportion of OHN (1.61 higher odds of OHN compared to the Midwest). This finding was consistent with the results of a study conducted by Paschal et al. (2016) [11], in which the outcome variable was unmet preventive dental needs.

Urbanicity also played a role in OHN among children with DD in our study. Higher odds of OHN were found among children with DD living in non-metropolitan areas (1.42, 95% CI: 1.02–1.99). This was consistent with what Skinner et al. (2006) found in a study that investigated the effect of rural residence on dental unmet need among CSHCN using 2005 NSCH [30]. They found that CSHCN who lived in rural areas were more likely to have unmet dental needs compared with their urban counterparts.

### Health insurance and poverty

Children with DD who were living in poverty and uninsured were more likely to have OHN, and this was consistent with the literature [3, 7, 8, 10, 31, 32]. A trend of increased OHN with an increased level of poverty was found in our study. Our results of higher odds of OHN with increasing levels of poverty were consistent with Nelson et al. [3], Lewis et al. [7], and Sannicandro et al. [10] regarding poor oral health and greater unmet needs for CSHCN from low-income families. We also found that public health insurance covered a large segment of children with DD (36.8%); nevertheless, the type of health insurance was not associated with increased odds of OHN. Our finding was consistent with Lewis et al. [7] who found that public insurance such as Medicaid and CHIP was not associated with unmet dental needs after adjusting for other confounding factors. McManus et al. [33] also confirmed no association between public health insurance eligibility and unmet preventive care needs.

### Extent of disability

Higher odds of OHN were found among children who were considerably affected by their condition. These results confirmed what has been reported in the literature regarding the association of condition severity/degree of the disability and OHN or unmet needs among CSHCN [4, 7, 34]. Our result was consistent with Sannicandro et al. finding that CSHCN who had a moderate or severe disability were more likely to have unmet dental needs [10]. Future research to identify, by a medical diagnosis, conditions that adversely affect the functional ability of children with DD is essential.

The study had several limitations. First, this cross-sectional study allowed us to examine associations but not causation, and temporal association was not determined. However, our findings illustrated valuable direction toward future research and targeted public health efforts toward prevention and intervention strategies for the severely affected subgroup of CSHCN. Second, many of our variables including the outcome variable “OHN” were collected through parents’ self-reported data, which were subject to various biases such as recall, reporting, and social desirability. No verification of oral health by calibrated examiners was conducted. Another limitation was that we used health insurance as a proxy for dental health insurance since there was no question in the survey about dental insurance. Generally, the percentage of children without dental insurance is twice that of children without medical insurance [35, 36]. Thus, using health insurance was a suboptimal substitute. Last, there was no verification of the parents’ reported diagnosis of DD among respondents to the survey. However, there is a notable consistency of the prevalence of individual DDs between the results of the NSCH and other nationally representative surveys, such as the National Health Interview Survey (NHIS) [20].

Our study had, however, multiple strengths. To our knowledge, this was the first study to measure the OHN of children with DD using a nationwide sample. Most studies investigated either an individual disorder or a broader group of CSHCN that included other medical conditions such as asthma, diabetes, blood disorders, and cancers. Although these conditions put children under the umbrella of special health care needs, they do not share a common risk of developmentally affected/delayed growth status. Second, our study also looked at the OHN of children with DD by geographic regions. Additionally, the NSCH included a large sample size of representative participants of children with DD from each state, which allowed us to perform robust analysis. Our findings could help policymakers focus efforts or target populations with the highest OHN by regions or to investigate factors related to the high OHN among these populations.

## Conclusion

Children with DD had more OHN than children without DD, and the more a child was affected by his/her condition, he/she were more likely to have OHN. We identified that being poor, uninsured, having a high level of disability, and living in the western United States were barriers for children with DD and were associated with higher odds of having OHN. Despite the high utilization rate of dental care services, children with DD still had poorer oral health than children without DD. The apparent disconnect between the utilization of dental services and commensurate outcomes suggests further research is needed. Advocacy efforts and policy changes are needed to develop affordable access that assesses, as early as possible, children with DD whose conditions impact their ability a great deal so that their potential OHN may be alleviated more effectively.

## Data Availability

The 2018 NSCH data are publicly available on the Census Bureau’s NSCH page (https://www.census.gov/data/datasets/2018/demo/nsch/nsch2018.html).

## References

[1] McPherson M, Arango P, Fox H, Lauver C, McManus M, Newacheck PW (1998). A new definition of children with special health care needs. Pediatrics.

[2] Frank M, Keels MA, Quinonez R, Roberts M, Divaris K (2019). Dental Caries Risk Varies Among Subgroups of Children with Special Health Care Needs. Pediatr Dent.

[3] Nelson LP, Getzin A, Graham D, Zhou J, Wagle EM, McQuiston J (2011). Unmet dental needs and barriers to care for children with significant special health care needs. Pediatr Dent.

[4] Iida H, Lewis C, Zhou C, Novak L, Grembowski D (2010). Dental care needs, use and expenditures among U.S. children with and without special health care needs. J Am Dent Assoc.

[5] Committee on Monitoring Access to Personal Health Care Services. Access to health care in America. National Academy Press; 1993.

[6] Norwood KW, Slayton RL (2013). Oral health care for children with developmental disabilities. Pediatrics.

[7] Lewis CW (2009). Dental care and children with special health care needs: a population-based perspective. Acad Pediatr.

[8] Sarkar M, Earley ER, Asti L, Chisolm DJ (2017). Differences in Health Care Needs, Health Care Utilization, and Health Care Outcomes Among Children With Special Health Care Needs in Ohio: A Comparative Analysis Between Medicaid and Private Insurance. J Public Health Manag Pract.

[9] Craig MH, Scott JM, Slayton RL, Walker AL, Chi DL (2019). Preventive dental care use for children with special health care needs in Washington's Access to Baby and Child Dentistry program. J Am Dent Assoc.

[10] Sannicandro T, Parish SL, Son E, Powell RM (2017). Health care changes for children with special health care needs, 2005–2011. Matern Child Health J.

[11] Paschal AM, Wilroy JD, Hawley SR (2016). Unmet needs for dental care in children with special health care needs. Prev Med Rep.

[12] Fisher K. Is there anything to smile about? A review of oral care for individuals with intellectual and developmental disabilities. Nurs Res Pract. 2012;2012. 10.1155/2012/860692 PMC3317070 22548164

[13] Centers for Disease Control and Prevention C. Facts About Developmental Disabilities 2019 [Available from: https://www.cdc.gov/ncbddd/developmentaldisabilities/facts.html.

[14] Chi DL, McManus BM, Carle AC (2014). Caregiver burden and preventive dental care use for US children with special health care needs: a stratified analysis based on functional limitation. Matern Child Health J.

[15] Chi DL (2018). Oral Health for US Children with Special Health Care Needs. Pediatr Clin North Am.

[16] Cheak-Zamora NC, Thullen M (2017). Disparities in Quality and Access to Care for Children with Developmental Disabilities and Multiple Health Conditions. Matern Child Health J.

[17] Ummer-Christian R, Iacono T, Grills N, Pradhan A, Hughes N, Gussy M (2018). Access to dental services for children with intellectual and developmental disabilities–A scoping review. Res Dev Disabil.

[18] Owens J, Dyer T, Mistry K (2010). People with learning disabilities and specialist services. Br Dent J.

[19] Dougall A, Fiske J (2008). Access to special care dentistry, part 1. Access British dental journal.

[20] Zablotsky B, Black LI, Maenner MJ, Schieve LA, Danielson ML, Bitsko RH, Blumberg SJ, Kogan MD, Boyle CA. Prevalence and trends of developmental disabilities among children in the United States: 2009–2017. Pediatrics. 2019;144(4). 10.1542/peds.2019-0811 PMC7076808 31558576

[21] Data Resource Center (DRC). About the National Survey of Children’s Health. Available from: https://www.childhealthdata.org/learn-about-the-nsch.

[22] Lipkin PH, Macias MM. Promoting Optimal Development: Identifying Infants and Young Children With Developmental Disorders Through Developmental Surveillance and Screening. Pediatrics. 2020;145(1). 10.1542/peds.2019-3449 31843861

[23] American Academy of Pediatrics. Council on children with disabilities, section on developmental behavioral pediatrics, Bright Futures Steering Committee, Medical Home Initiatives For Children With Special Needs Project Advisory Committee. Identifying infants and young children with developmental disorders in the medical home: An algorithm for developmental surveillance and screening. Pediatrics. 2006;118(1):405–20. 10.1542/peds.2006-1231 16818591

[24] The United States Office of Management and Budget. 2010 Standards for Delineating Metropolitan and Micropolitan Statistical Areas; Notice; 2010. Available from: https://www.govinfo.gov/content/pkg/FR-2010-06-28/pdf/2010-15605.pdf.

[25] U.S. Department of Commerce Economics and Statistics Administration U.S. Census Bureau. Census Regions and Divisions of the United States; 2010. Available from: https://www2.census.gov/geo/pdfs/maps-data/maps/reference/us_regdiv.pdf.

[26] Jaber MA (2011). Dental caries experience, oral health status and treatment needs of dental patients with autism. J Appl Oral Sci.

[27] Moreira RN, Alcântara CEP, Mota-Veloso I, Marinho SA, Ramos-Jorge ML, Oliveira-Ferreira F (2012). Does intellectual disability affect the development of dental caries in patients with cerebral palsy?. Res Dev Disabil.

[28] Shyama M, Al-Mutawa SA, Morris RE, Sugathan T (2001). Dental caries experience of disabled children and young adults. Community Dent Health.

[29] US Department of Health and Human Services. Title V maternal and child health services block grant program. Retrieved April. 2017;7.

[30] Skinner AC, Slifkin RT, Mayer ML (2006). The effect of rural residence on dental unmet need for children with special health care needs. J Rural Health.

[31] Berdahl T, Hudson J, Simpson L, McCormick MC (2016). Annual report on Children's health care: dental and orthodontic utilization and expenditures for children, 2010–2012. Acad Pediatr.

[32] Lee JN, Scott JM, Chi DL. Oral health behaviours and dental caries in low‐income children with special healthcare needs: A prospective observational study. Int J Paediatr Dent. 2020;30(6):749–57. 10.1111/ipd.12656 PMC11682719 32306501

[33] McManus BM, Chi D, Carle A (2016). State Medicaid Eligibility Criteria and Unmet Preventive Dental Care Need for CSHCN. Matern Child Health J.

[34] McKinney CM, Nelson T, Scott JM, Heaton LJ, Vaughn MG, Lewis CW (2014). Predictors of unmet dental need in children with autism spectrum disorder: results from a national sample. Acad Pediatr.

[35] Nasseh K, Vujicic M. Dental benefits coverage rates increased for children and young adults in 2013. Health Policy Institute Research Brief. American Dental Association. 2015.

[36] Vujicic M, Buchmueller T, Klein R (2016). Dental care presents the highest level of financial barriers, compared to other types of health care services. Health Aff.

